# The complete mitochondrial genome of northern grasshopper mouse (*Onychomys leucogaster*)

**DOI:** 10.1080/23802359.2017.1347905

**Published:** 2017-07-07

**Authors:** Guiqi Bi

**Affiliations:** a Key Laboratory of Marine Genetics and Breeding (OUC), Ministry of Education, Qingdao, P.R. China;; b College of Marine Life Sciences, Ocean University of China, Qingdao, P.R. China

**Keywords:** *Onychomys leucogaster*, mitogenome, phylogenetic analysis

## Abstract

In this study, we presented the complete mitochondrial genome of northern grasshopper mouse (*Onychomys leucogaster*). The circular genome is 16,370 bp in length and contains 13 protein-coding genes (PCGs), 22 transfer RNA (tRNA) genes, 2 ribosome RNA genes, and 1 D-loop control regions. The overall nucleotide composition is: 31.2% A, 25.2% T, 29.9% C, and 13.6% G, with a total G + C content of 43.53%. The phylogenetic tree was constructed to validate the taxonomic status of *Onychomys leucogaster*, exhibiting a closest relationship with *Neotoma fuscipes*.

The northern grasshopper mouse (*Onychomys leucogaster*), which is widely distributed in grassland and shrub steppe habitats, is a North American carnivorous rodent of the family Cricetidae (Wilson & Reeder 2005). The *O. leucogaster* has many unique features when compared with other rodent species. For example, they are highly aggressive and territorial (Ruffer 1968) and have large home ranges (Blair 1953). And they also have a complex communication system (Ruffer 1966). In 2008, the grasshopper mouse is listed as Least Concern (LC) on the IUCN Red List of Threatened Species. But, little molecular genetic studies have been done regarding this species. Consequently, the various DNA information of *O. leucogaster* is still limited. In the present study, the complete mitogenome sequence of *O. leucogaster* has been determined. The work provides the reference sequence of mitogenome for *O. leucogaster* that may be utilized for the determination of population genetic studies in the future.

The specimen was collected from the Rio Grande Plains of South Texas, USA. The muscle tissue and total genomic DNA that was extracted through Animal Tissues Genomic DNA Extraction Kit (Solarbio, BJ, CN) were stored in the sequencing company (BGI Tech, Shenzhen, China). Total genomic DNA of muscle tissue was extracted through Animal Tissues Genomic DNA Extraction Kit (Solarbio, BJ, CN). Purified DNA was fragmented and used to construct the sequencing libraries following the instructions of NEBNext^®^ Ultra™ II DNA Library Prep Kit (NEB, BJ, CN). Whole genomic sequencing was performed by the Illumina HiSeq 2500 Sequencing Platform (Illumina, Hayward, CA). Adapters and low-quality reads were removed using the NGS QC Toolkit (Patel & Jain 2012). The mitochondrial reads from pre-filtered reads were screened out by bowtie2 (Langmead & Salzberg 2012) using other rodent mitochondrial genomes as references. Then, assembly as implemented by SPAdes 3.9.0 (Bankevich et al. 2012). Gaps among contigs were filled using MITObim V1.9 (Hahn et al. 2013). The determined genome was annotated using the MFannot tool (http://megasun.bch.umontreal.ca/cgi-bin/mfannot/mfannotInterface.pl), combined with manual corrections. tRNAs were annotated by ARWEN Web Server (Laslett & Canbäck 2008).

The complete mitogenome of *Onychomys leucogaster* (GenBank accession: NC_029760) is a closed-circular molecule of 16,370 bp bp in length, which is well within the size range observed in the completely sequenced other Xenarthrans mitogenomes. It presents the typical set of 37 genes observed in metazoan mitogenomes, including 13 PCGs (*cox*1-3, *co*b, *nad*1-6, *nad*4L, *atp*6 and *atp*8), 22 tRNA genes, 2 genes for ribosomal RNA subunits (*rrn*S and *rrn*L), and 1 D-loop control regions. The overall nucleotide composition is: 34.7% A, 24.4% T, 12.9% C, and 28.0% G, with a total G + C content of 37.33%.

To validate the phylogenetic position of *Onychomys leucogaster*, the genome-wide alignment of 24 rodent mitochondrial genomes was constructed by HomBlocks (https://github. com/fenghen360/HomBlocks), resulting in 15,126 characters of each species, which including all PCGs and rRNA genes. The JMODELTEST 2.0.2 (Darriba et al. 2012) was used to ascertain the best-ft model of nucleotide substitution for sequences with Bayesian information criterion (BIC). Bayesian analysis (BI) and maximum likelihood (ML) were used to reconstruct the phylogenetic trees. Bayesian phylogenetic analysis was conducted with MrBayes 3.2.5 (Ronquist et al. 2012) based on the most appropriate model. Four Markov chains were run for 10,000,000 generations to estimate the posterior probability (PP) distribution (sampling 1 tree with 100 replicates for each run). After discarding the frst 2000 trees as burn-in that was referred to log likehood values, 50% majority-rule consensus trees were estimated for the remaining trees. ML analysis was performed using RAxML (Stamatakis 2014) with the GTR + G + I model. Bootstrap values were calculated using 1000 replicates to assess the node support. Phylogenetic relationships obtained with the ML approach were identical to those obtained using the BI. As shown in the phylogenetic tree (Figure 1), the mt genome reported here exhibited the closest relationship with Dusky-footed woodrat (*Neotoma fuscipes*:GenBank accession number: KU745736.1).

**Figure 1.**
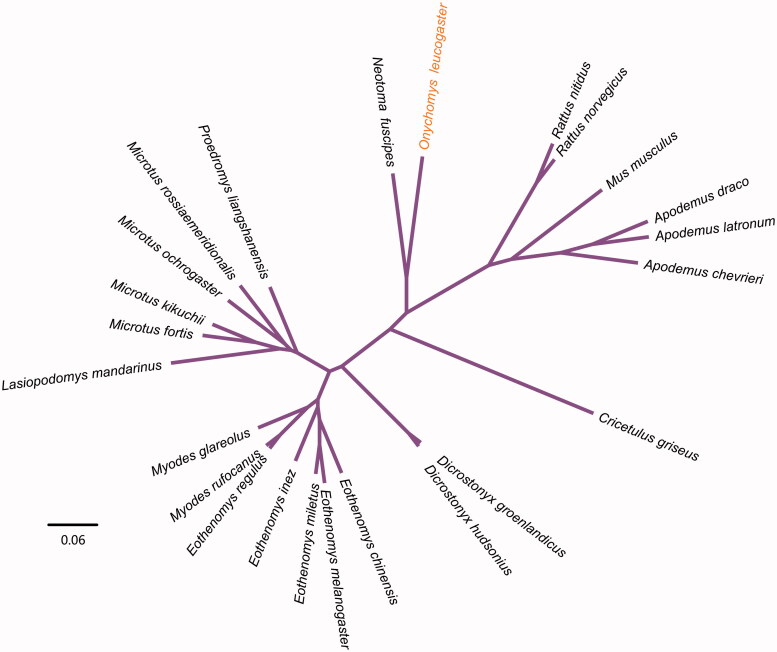
Phylogenetic relationships among 24 rodent mt genoems. This tree was drawn without setting of a outgroup. All nodes exhibit full posterior probability (PP) and 100% RAxML supported bootstrap. The length of branch represents the divergence distance. Mitogenome accession numbers used in this phylogeny analysis: *Apodemus chevrieri* (HQ896683.1), *Apodemus draco* (HQ333255.1), *Apodemus latronum* (HQ333256.1), *Cricetulus griseus* (EU660217.1), *Dicrostonyx groenlandicus* (KX712239.1), *Dicrostonyx hudsonius* (KX683880.1), *Eothenomys chinensis* (FJ483847.1), *Eothenomys inez* (KU200225.1), *Eothenomys melanogaster* (KP997311.1), *Eothenomys miletus* (KX014874.1), *Eothenomys regulus* (JN629046.1), *Lasiopodomys mandarinus* (JX014233.1), *Microtus fortis* (JF261174.1), *Microtus kikuchii* (Microtus kikuchii), *Microtus ochrogaster* (KT166982.1), *Microtus rossiaemeridionalis* (DQ015676.1), *Mus musculus* (KF781655.1), *Myodes glareolus* (KF918859.1), *Myodes rufocanus* (KT725595.1), *Neotoma fuscipes* (KU745736.1), *Proedromys liangshanensis* (FJ463038.1), *Rattus nitidus* (KX058347.1), *Rattus norvegicus* (FJ919759.1).
